# Microwave ablation enhances tumor-specific immune response in patients with hepatocellular carcinoma

**DOI:** 10.1007/s00262-020-02734-1

**Published:** 2020-10-02

**Authors:** Katharina Leuchte, Elena Staib, Martin Thelen, Philipp Gödel, Axel Lechner, Peter Zentis, Maria Garcia-Marquez, Dirk Waldschmidt, Rabi Raj Datta, Roger Wahba, Christian Wybranski, Thomas Zander, Alexander Quaas, Uta Drebber, Dirk Ludger Stippel, Christiane Bruns, Michael von Bergwelt-Baildon, Kerstin Wennhold, Hans Anton Schlößer

**Affiliations:** 1 Center for Molecular Medicine Cologne, University of Cologne, Köln, Germany; 2 Department I of Internal Medicine and Center for Integrated Oncology (CIO) Aachen Bonn Cologne Duesseldorf, University Hospital Cologne, Kerpener Straße 62, 50937 Köln, Germany; 3 Department of Otorhinolaryngology, Head and Neck Surgery, University Hospital, LMU Munich, München, Germany; 4 Cluster of Excellence in Aging-Associated Disease, Core Facility Imaging, University of Cologne, Köln, Germany; 5 Department of Gastroenterology and Hepatology, University Hospital Cologne, Köln, Germany; 6 Department of General, Visceral and Cancer Surgery, University Hospital Cologne, Köln, Germany; 7 Department of Diagnostic and Interventional Radiology, University Hospital Cologne, Köln, Germany; 8 Institute of Pathology, University Hospital Cologne, Köln, Germany; 9 German Cancer Consortium (DKTK), Heidelberg, Germany; 10 Department of Internal Medicine III, University Hospital, LMU Munich, München, Germany

**Keywords:** Immunogenic cell death, Immunotherapy, Antigens, Abscopal effect

## Abstract

Thermal ablative therapies are standard treatments for localized hepatocellular carcinoma (HCC). In addition to local tumor destruction, ablation leads to abscopal effects in distant lesions most likely mediated by an anti-tumor immune response. Although microwave ablation (MWA) is increasingly substituting other ablative techniques, its systemic immunostimulatory effects are poorly studied. We analyzed tumor-specific immune responses in peripheral blood of HCC patients after thermal ablation with regard to T cell responses and disease outcome. While comprehensive flow cytometric analyses in sequential samples of a prospective patient cohort (*n* = 23) demonstrated only moderate effects of MWA on circulating immune cell subsets, fluorospot analyses of specific T cell responses against seven tumor-associated antigens (TTAs) revealed de-novo or enhanced tumor-specific immune responses in 30% of patients. This anti-tumor immune response was related to tumor control as Interferon-y and Interleukin-5 T cell responses against TAAs were more frequent in patients with a long-time remission (> 1 year) after MWA (7/16) compared to patients suffering from an early relapse (0/13 patients) and presence of tumor-specific T cell response (IFN-y and/or IL-5) was associated to longer progression-free survival (27.5 vs. 10.0 months). Digital image analysis of immunohistochemically stained archival HCC samples (*n* = 18) of patients receiving combined MWA and resection revealed a superior disease-free survival of patients with high T cell abundance at the time of thermal ablation (37.4 vs. 13.1 months). Our data demonstrates remarkable immune-related effects of MWA in HCC patients and provides additional evidence for a combination of local ablation and immunotherapy in this challenging disease.

**Electronic supplementary material:**

The online version of this article (10.1007/s00262-020-02734-1) contains supplementary material, which is available to authorized users.

## Introduction

Hepatocellular carcinoma (HCC) is the second leading cause of cancer-related deaths worldwide [1]. In addition to surgery and chemotherapy, thermal ablative therapies are routinely used and included in common guidelines for localized HCC [2, 3]. Microwave ablation (MWA), which has entered the field a decade ago, is increasingly used and rapidly replaces radiofrequency ablation (RFA) [4]. Shorter ablation times and potentially larger ablation zones are key advantages of MWA. Local tumor control and overall survival following RFA and MWA seem to be comparable [5]. Abscopal effects, defined as regression of distant, untreated lesions, have been described for both treatments [6–8]. Previous publications suggest induction or enhancement of systemic anti-tumor immune responses induced by the destruction of HCC [4, 9–11]. Released antigens are processed and presented by antigen-presenting cells, which enhance or induce anti-tumor T cell response [12, 13]. A similar mechanism has been described for melanoma in a study showing an abscopal effect following combined treatment with radiotherapy and Ipilimumab [14]. Immune stimulatory effects induced by thermoablation in HCC have mainly been investigated for RFA [12]. Following RFA, a specific T cell response with increased proportions of circulating activated T cells, natural killer (NK) cells and production of tumor-specific antibodies is triggered [15, 16]. In a larger clinical study with 69 HCC patients, Mizukoshi et al. identified specific T cell responses to tumor-associated antigens (TAA) in 62% of cases. The number of TAA-specific T cells correlated with survival. Notably, the specific T cell response had a limited lifespan and was significantly decreased at 24 weeks after RFA [17]. Immune-stimulatory effects of MWA have been rarely investigated. An increase of T cells, NK cells and macrophages could be demonstrated in HCC lesions treated with MWA [18]. Two other studies provided the first evidence for a systemic effect of MWA on circulating T cell subsets [19, 20]. We used flow cytometry, fluorospot analyses of tumor-specific T cell responses and digital pathology to determine systemic immune-modulatory effects of MWA and immune correlates of response to thermal ablation.

## Materials and methods

### Patient characteristics and cell isolation from human peripheral blood

Sequential blood samples (prior to intervention, day 7 and day 90) of 23 HCC patients undergoing MWA and single blood samples of 30 patients with early relapse (< 1 year; *n* = 13) or long-term remission (> 1 year; *n* = 17) were collected between 2016 and 2018. Peripheral blood mononuclear cells (PBMC) were purified using density-based separation (PAN-Biotech) by centrifuging for 20 min at 1080×*g*. Tumor samples of additional 18 patients, who underwent combined treatment with surgical resection of one lesion and MWA of a second lesion were obtained for immunohistochemical analyses. The patient characteristics are summarized in Table 1. All patients gave written informed consent and our local ethics committee approved this study (No. 11-116; 17-282).

**Table 1:** Patient characteristics

	Prospective patients	Patients with combination of ablation and surgical resection	Relapsed patients	Patients in long-term remission	*p* value
	Number (%)	Number (%)	Number (%)	Number (%)	
	23	18	13	17	
Age median [range, years]	65 [48–83]	70 [41–80]	70 [55–81]	71 [50–87]	0.78
Sex					1.0
Female	7 (30.43%)	7 (38.89%)	2 (15.38%)	3 (17.65%)	
Male	16 (69.57%)	11 (61.11%)	11 (84.62%)	14 (82.35%)	
Etiology					0.86
Chronic Hepatitis B	2 (8.70%)	4 (22.22%)	2 (15.38%)	5 (29.41%)	
Chronic Hepatitis C	7 (30.43%)	5 (27.78%)	3 (23.08%)	2 (11.76%)	
Alcohol abuse	3 (13.04%)	3 (16.67%)	2 (15.38%)	2 (11.76%)	
Several risk factors	7 (30.43%)	0 (0.00%)	1 (7.69%)	2 (11.76%)	
Other	4 (17.39%)	6 (33.33%)	5 (38.46%)	6 (35.29%)	
BCLC Stage					0.79
0	1 (4.35%)	0 (0.00%)	0 (0.00%)	0 (0.00%)	
A	15 (65.22%)	5 (27.78%)	10 (76.92%)	14 (82.35%)	
B	3 (13.04%)	11 (61.11%)	2 (15.38%)	3 (17.65%)	
C	4 (17.39%)	2 (11.11%)	1 (7.69%)	0 (0.00%)	
D	0 (0.00%)	0 (0.00%)	0 (0.00%)	0 (0.00%)	
MELD median [range]	7 [6–14]	7 [6–11]	7.5 [6–17]	7 [6–10]	0.72
Tumor diameter					0.33
< 3 cm	16 (69.57%)	7 (38.89%)	9 (69.23%)	13 (76.47%)	
3–5 cm	7 (30.43%)	5 (27.78%)	4 (30.77%)	2 (11.76%)	
> 5 cm	0 (0.00%)	6 (33.33%)	0 (0.00%)	2 (11.76%)	
Tumor volume median [range, cm^3^]	3.5 [0.1–30.1]	n.a	2.1 [0.1–14.1]	1.8 [0.1–48.3]	0.44
Ablation:tumor ratio mean	3.3	n.a	3.6	3.5	0.93
Ablation energy median [range, kJ]	50 [24–120]	50.6 [25.2–84]	53.6 [12–122.6]	55.2 [15–90.4]	0.90
Previous treatment					0.29
Local ablative therapy	4 (17.39%)	1 (5.56%)	2 (15.38%)	4 (23.53%)	
Surgical resection	3 (13.04%)	4 (22.22%)	2 (15.38%)	1 (5.88%)	
TACE/TAE/Radiotherapy	2 (8.70%)	0 (0.00%)	3 (23.08%)	0 (0.00%)	
None	14 (60.87%)	13 (72.22%)	6 (46.15%)	12 (70.59%)	
AFP					0.36
< 5 [kIU/l]	7 (30.43%)	2 (11.11%)	1 (7.69%)	1 (5.88%)	
≥ 5 [kIU/l]	5 (21.74%)	13 (72.22%)	2 (15.38%)	7 (41.18%)	
Not determined	11 (47.83%)	3 (16.67%)	10 (76.92%)	9 (52.94%)	

### Computed tomography-guided MWA

CT-guided MWA procedures were performed employing a 256-row multi-slice scanner (iCT 256, Philips Healthcare, Amsterdam, Netherlands) under general anesthesia. Planning datasets were acquired in arterial and portal venous phase after intravenous administration of non-ionic, iodinated contrast media (Accupaque 350 mg/ml, GE Healthcare). The MWA system consisted of a 2.45-GHz generator generating a maximum of 140 W and a water-cooled 14G-antenna (Amica). Placement of the microwave antenna within the target lesion was performed under CT fluoroscopy by one experienced interventional radiologist. Ablation was performed according to the manufacturer’s recommendations with the aim to generate a safety margin of at least 0.5 cm. Complete necrosis after MWA was confirmed by dynamic contrast-enhanced CT or MRI and ablation-to-tumor ratio was calculated for each patient (Table 1).

### Flow cytometry

At least 10 × 10^5^ events per sample were acquired (Gallios, Beckman Coulter). Phenotypic characterization of T, B and NK cell subsets was performed using a panel of fluorochrome-labeled antibodies (Supplementary Table 1). Cells were fixated and permeabilized using a fix and/or perm buffer before incubation with antibodies for intracellular staining of FoxP3. Analysis was performed using Kaluza software (Beckman Coulter). Gating strategy is shown in Supplementary Fig. 2.

### Immunohistochemistry, IS and TB Score

Immunohistochemical staining was performed on formalin-fixed, paraffin-embedded tissue sections (3 μm). Following deparaffinization in alcohol, antigen retrieval (20 min 90 °C) was performed in citrate buffer (pH = 6.0). Endogenous peroxidase activity was blocked with peroxidase methanol solution. Primary (Supplementary Table 1) and appropriate secondary antibodies (Dako EnVision; K4001, K4009) were applied. The sections were incubated with 3-amino-9-ethylcarbazol (AEC-substrate solution; Dako) for 15 min (CD3), 30 min (CD8, FoxP3), 10 min (CD20, CD4). We included tonsillar and lymph node tissue as positive and normal liver tissue as negative controls. Hematoxylin was used as nuclear counterstaining. The stained sections were scanned on a slidescanner (SCN400, Leica Microsystems) at 40 × magnification. Cells were quantified using Imagescope, KNIME, ILASTIK and Cell Profiler softwares (Supplementary Fig. 3). Central tumor (CT) and Invasive margin (IM: area ranging from 50 μm within to 300 μm outside the tumor) areas were marked (Drebber, U). High infiltration was defined as cell density above the median. The Immunoscore (IS) and the TB score were calculated [21, 22].

### Fluorospot

0.2 × 10^6^ PBMC were incubated with peptide pools of the cancer-testis antigens MAGE-A1, MAGE-A3, MAGE-A4, MAGE-C1, NY-ESO-1, PRAME, AFP (all JPT) on precoated anti-human 3-color IFN-y, IL-5 and IL-10 Fluorospot plates (Mabtech) for 20 h. AIM-V medium was supplemented with 0.1 μg/mL anti-CD28 (Mabtech). An antibody to CD3 (Mabtech) as well as CEFX Ultra SuperStim Pool (JPT) were used as positive controls in a dilution of 1:1000 and 1 µg/mL, respectively. Fluorochrome-labeled antibodies anti-BAM-490, SA-550 and anti-WASP-640 (all Mabtech) were used to visualize IFN-y, IL-10 and IL-5 cytokine production. Fluorospot analysis was performed on an AID iSpot Spectrum.

### Data analysis

Unless otherwise stated, data are shown as mean ± standard error of the mean. Flow cytometry data were analyzed using Kaluza Software (Version 2.1, Beckman Coulter) and GraphPad Prism 8 (GraphPad Software). Repeated measures one-way ANOVA was performed to compare independent measures of more than two groups, student’s *t* test for comparison of two groups. Fisher’s exact test was applied to test for association between categorical variables. Survival differences of Kaplan–Meier curves were analyzed using log-rank test. *P* values < 0.05 were considered statistically significant.

## Results

### MWA treatment and clinical parameters

The epidemiological, clinical and pathological characteristics of included patients are depicted in Table 1 and did not show significant differences between patients with early relapse and patients in long-term remission. MWA was performed according to our local guidelines with highly standardized protocols. Ablation energies depended on the tumor size and did not differ between the groups. The mean ablation:tumor ratio was 3.3 for prospective patients, 3.6 for patients with early relapse and 3.5 for patients with long-term remission (*p* = 0.93). Calculation of the ratios is shown in Supplementary Fig. 1. The majority of our patients was included at first diagnosis. For pretreated patients, the mean interval between the last previous treatment and MWA was 420 days ± 50.5 (Supplementary Fig. 5a).

### Impact of MWA treatment on circulating B and T cell subsets

**Fig. 1:**
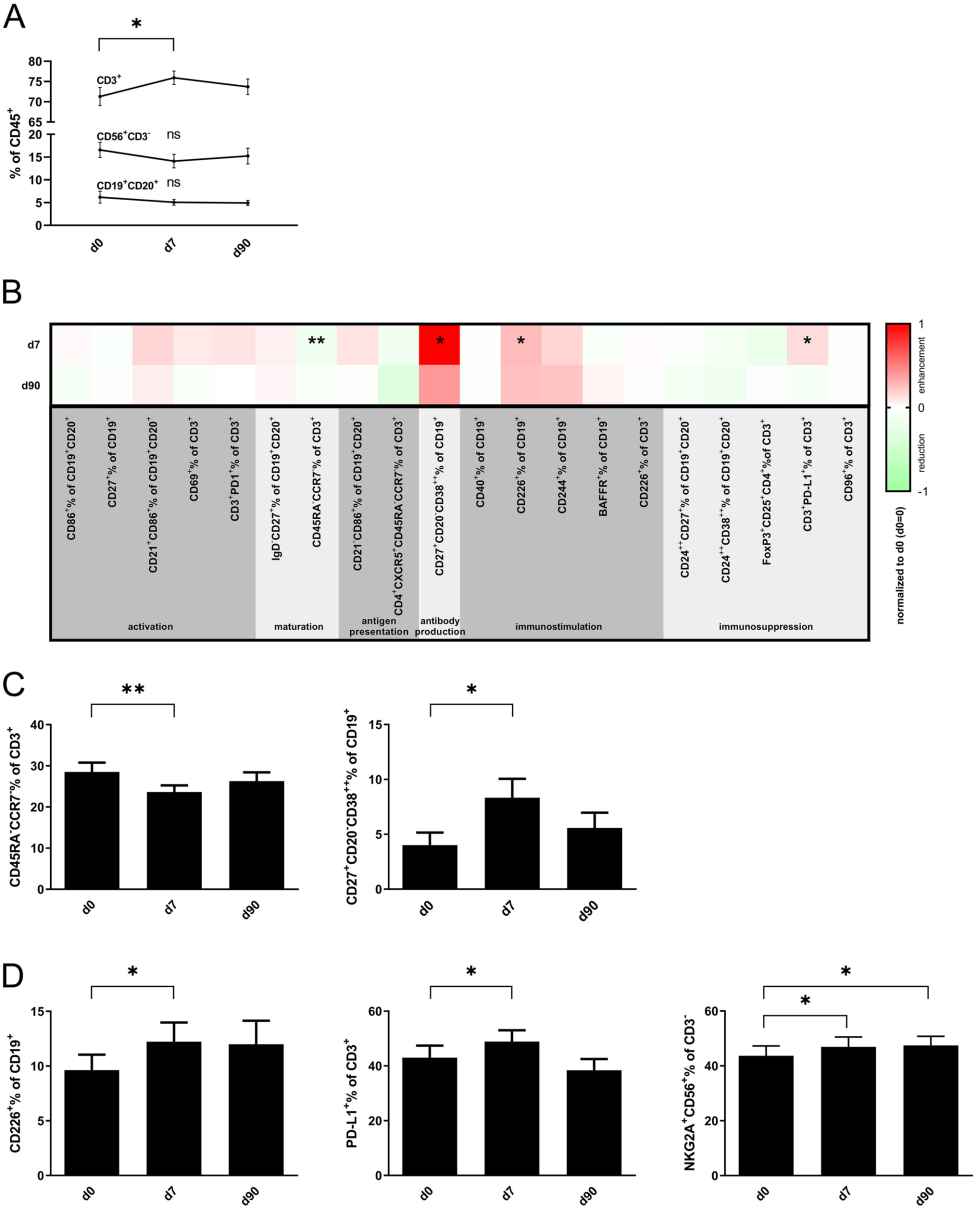
Lymphocytic subsets of prospective patients: **a** PBMC of MWA-treated patients before (d0), 7 (d7) and 90 (d90) days after intervention (*n* = 20) were analysed by flow cytometry for the percentages of T, B and NK cells. **b** Heatmap showing an overview of analyzed lymphocytic subsets normalized to results obtained from PBMC of d0 (PBMC d0 = 0). **c** T, B and NK cell subsets in PBMC of MWA-treated patients at d0, d7 d90 (*n* = 20) were analyzed by flow cytometry for the percentages of CD45RA^−^CCR7^−^CD3^+^ effector memory T cells and CD27^+^CD20^−^CD38^++^CD19^+^ plasmablasts, **d** CD226^+^CD19^+^ B cells, PD-L1^+^CD3^+^ T cells, and NKG2A^+^CD56^+^ NK cells

We used comprehensive 10-color flow cytometry to characterize changes in lymphocyte subsets in PBMC of 23 HCC patients prior to, 7 days and 90 days after MWA. The percentage of CD3^+^ T cells increased on day 7 after MWA (75.9% ± 1.7 vs. 71.3% ± 2.2 on day 0; *p* = 0.02), while CD19^+^CD20^+^ B cells and CD56^+^CD3^−^ NK cells remained stable (Fig. 1a). We analyzed specific phenotypes of functionally distinct T and B cell subsets (activation, maturation, antigen presentation, antibody production, immune stimulation and immunosuppression) (Fig. 1b). The majority of T and B cell subsets remained unchanged after MWA treatment. The frequency of effector memory T cells (CCR7^−^CD45RA^−^ in % of CD45^+^CD3^+^) was reduced 7 days following MWA (23.7% ± 1.6, day 0 28.5% ± 2.3; *p* < 0.01). Plasmablasts (CD27^+^CD20^−^CD38^++^ in % of CD19^+^) were increased on day 7 after treatment (8.3% ± 1.7, day 0 4.0% ± 1.2; *p* = 0.02) (Fig. 1c). Percentages of lymphocytes expressing costimulatory or coinhibitory cells were mostly unchanged, except for the costimulatory molecule CD226^+^ (DNAM-1) on CD19^+^ B cells, increasing from 9.6% ± 1.4 on day 0 to 12.2% ± 1.8 on day 7, *p* = 0.03, and 12.0% ± 2.1 on day 90 (*p* = 0.60). The percentage of PD-L1-expressing CD3^+^ T cells was increased on day 7 (48.9% ± 4.1; day 0 43.0% ± 4.4, *p* = 0.02; day 90 38.5% ± 4.1, *p* = 0.06). NKG2A^+^CD56^bright^ NK cells, representing a less differentiated NK cell subset capable of antigen-dependent cytokine production and proliferation, were enduringly increased after MWA treatment (day 0 43.7% ± 3.6, day 7 46.9% ± 3.6; *p* = 0.02, and day 90 47.5% ± 3.3; *p* = 0.02) (Fig. 1d). The frequency of activated NK cells (CD25^+^%CD56^+^) remained unchanged following MWA treatment (day 0 0.20% ± 0.04 vs. day 7 0.22% ± 0.05 vs. day 90 0.21% ± 0.06; Supplementary Fig. 6b).

### Enhancement of preexisting and induction of de novo TAA-specific T cell responses after MWA treatment can be frequently detected after MWA treatment

**Fig. 2:**
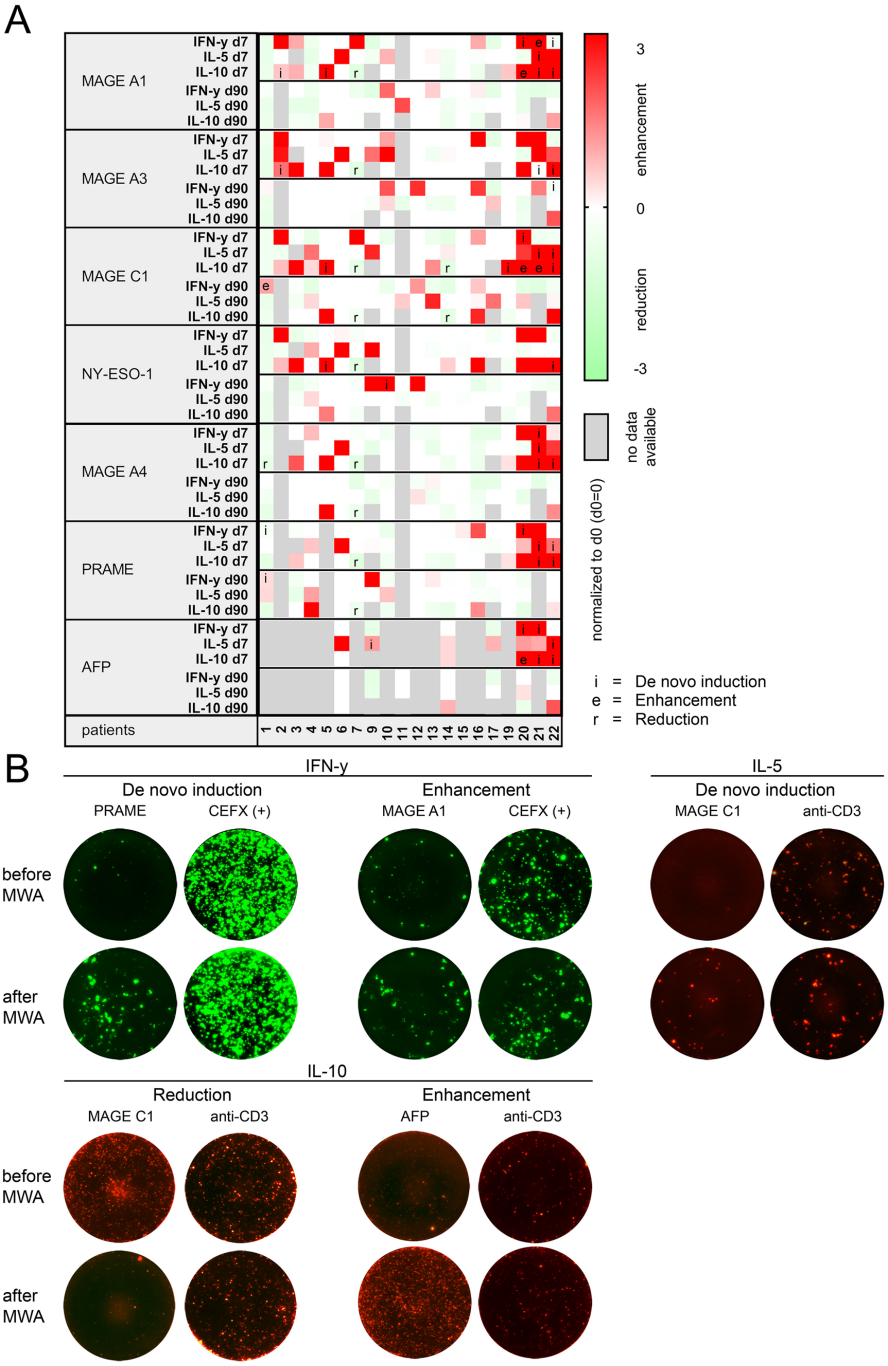
Analysis of the tumor antigen-specific T cell response by Fluorospot analysis: **a** Heatmap showing an overview of immune responses against HCC-specific tumor antigens (MAGE-A1, MAGE-A3, MAGE-C1, NY-ESO-1, MAGE-A4, PRAME, AFP) after MWA (d7, d90) normalized to specific spots obtained from PBMC on d0 (specific spots d0 = 0) (*n* = 20), pointing out significant de novo inductions (i) and enhancements (e) of IFN-y, IL-5 or IL-10 response and significant reductions (r) of IL-10 production. **b** Exemplary Fluorospot analysis of de novo induction of IFN-y response against PRAME (patient 20), enhancement of IFN-y response against MAGE-A1 (patient 21), De-novo induction of IL-5 response against CTA MAGE C1 (patient 21), reduction of IL-10 response against CTA MAGE C1 (patient 14), enhancement of IL-10 response against tumor antigen AFP (patient 20). Positive controls (CEFX for IFN-y, anti-CD3 for IL-5 and IL-10) for each patient are depicted on the right

We screened PBMC of 23 patients before, 7 days and 90 days after MWA for circulating T cells specific for TAAs frequently described in HCC [23, 24]. Based on literature and common databases, we defined a panel including MAGE-A1, MAGE-A3, MAGE-A4, MAGE-C1, NY-ESO-1, PRAME and AFP for Fluorospot analyses of IFN-y, IL-5 and IL-10 secretion. As PBMC were limited, we reduced the number of TAAs in some cases. 9 IL-10 samples and 2 IL-5 analyses were excluded due to failure of the anti-CD3 positive control (Supplementary Fig. 4). Unspecific immune activation caused by MWA-induced inflammatory signals could be ruled out by testing against an independent peptide pool (CEFX) (*p* = 0.72; Supplementary Fig. 4). 5 out of 20 patients (25%) developed IFN-y responses against at least one TAA (patient 1, 10, 20, 21 and 22; Fig. 2a and representative pictures Fig. 2b). All of those patients developed de novo responses, which were not detected in pretreatment samples. In two cases our analyses revealed an additional enhancement of preexisting TAA-specific responses (patient 1 and 21; Fig. 2a). Overall, IFN-y responses were induced against all of the 7 TAAs, with MAGE-C1 being the most frequently recognized antigen (4/20, 20%). Simultaneous responses to multiple TAAs were seen in 3 patients. Out of these, 2 patients showed IFN-y responses against more than 3 TAAs and 1 against 3 TAAs. Regarding the T helper cell 2-associated cytokine IL-5, we detected de novo induction of peptide-specific responses in 3 out of 19 patients (15.8%; patient 9, 21 and 22; Fig. 2a and b). No enhancement of IL-5 responses was observed. Simultaneous IL-5 responses to multiple TAAs were seen in 2 patients. Out of these, 1 patient showed IL-5 responses against 3 TAAs and 1 against 4 TAAs. TAA-specific secretion of IL-10, the third cytokine included in these analyses, could be detected in pretreatment samples of 5/20 patients (25%). A significant decrease of IL-10 secreting T cells was observed in 3/17 (17.6%; patients 1, 7, 14) and a significant increase in 2/17 (11.8%; patients 20, 21; Fig. 2a and b). De novo induction of TAA-specific IL-10 secretion occurred in 6/17 patients after MWA (35.3%; patients 19, 5, 2, 20, 22, 21). With regard to the time course, the majority of patients showed a transient T cell response on day 7 and reduced reactivity on day 90 for all analyzed cytokines (IFN-y: 3/20, 15%; patient 20, 21, 22, IL-5: 2/18, 11.1%; patient 9, 22). However, 2/20 patients (10%) showed IFN-y secretion only on day 90 (patient 10 and 22), one patient (5%) showed a persistent T cell reactivity after MWA (patient 1; Fig. 2a). For Th2 responses, the pattern was similar with 3/19 transient responses on day 7 (15.8%; patient 9, 21 and 22; Fig. 2a). Taken together, 6 out of 20 patients (30%) showed TAA-specific secretion of IFN-y or IL-5 upon stimulation with HCC-associated antigens after MWA.

### Increase of activated T and B cell subsets in patients with long-term remission after MWA

**Fig. 3:**
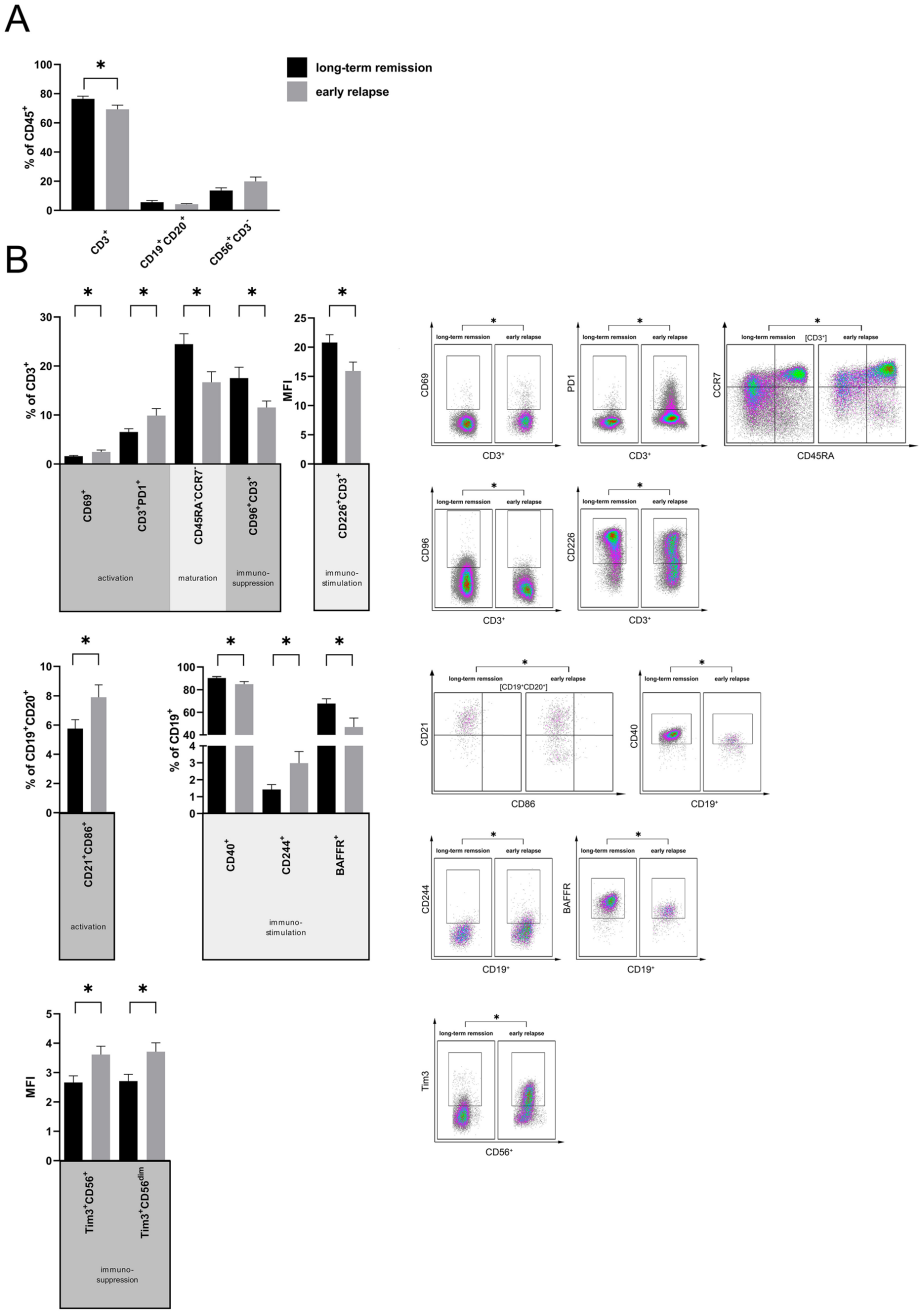
Lymphocytic subsets of retrospective patients. Retrospective MWA-treated patients were divided into two cohorts, patients in long-term remission (> 1 year; *n* = 17) and patients with an early relapse (< 1 year; *n* = 13). **a** PBMC of both cohorts were analysed by flow cytometry for the percentages of T, B and NK cells. **b** T cell subsets in PBMC of both cohorts were analyzed by flow cytometry for the percentages of CD69^+^CD3^+^ T cells, PD1^+^CD3^+^ T cells, CD45RA^−^CCR7^−^CD3^+^ effector memory T cells, CD96^+^CD3^+^ T cells and MFI of CD226^+^CD3^+^ T cells. B cell subsets in PBMC were examined by flow cytometry for the percentages of CD86^−^CD21^−^CD19^+^CD20^+^ B cells, CD40, CD244 and BAFF-R expressing CD19^+^ B cells. CD56^+^ NK cells were investigated for expression of Tim-3. Exemplary FACS plots on the right illustrate significant differences between relapsed patients and patients in remission

To assess whether the long-term response to MWA is associated with specific changes in circulating immune cell subsets, we analyzed PBMC of patients with early relapse (< 1 year, *n* = 13) and patients in long-term remission (> 1 year, *n* = 17). We stained for the same set of lineage markers, activation markers and immune checkpoints as described above. CD45^+^CD3^+^ T cells accounted for the majority of PBMC in both groups and were decreased in patients with early relapse (70.5% ± 3.1) compared to patients in long-term remission (77.7% ± 1.8, *p* = 0.04). Percentages of CD45^+^CD19^+^CD20^+^ B cells and CD45^+^CD56^+^CD3^−^ NK cells did not show differences (Fig. 3a). T cells expressing the activation marker CD 69 were enriched in relapsed patients compared to patients with long-term remission (CD69^+^ in % of CD3^+^: 2.5% ± 0.4 vs. 1.6% ± 0.2, *p* = 0.04; Fig. 3b). The T cell exhaustion marker PD-1 was also upregulated in relapsed patients (PD-1^+^ in % of CD3^+^: 9.9% ± 1.4 vs. 6.5% ± 0.7, *p* = 0.03; Fig. 3b). In disease-free patients we identified a higher proportion of circulating effector memory T cells (CD45RA^−^CCR7^−^ in % of CD3^+^) (24.5% ± 2.1 vs. 16.7% ± 2.2, *p* = 0.02), higher costimulatory molecule CD226 (DNAM-1) expression (MFI: 20.8 ± 1.3 vs. 15.9 ± 1.5, *p* = 0.02) and a higher frequency of CD96-expressing T cells (17.5% ± 2.2 vs. 11.5% ± 1.3, *p* = 0.04) (Fig. 3b, top). Analysis of B cell subsets revealed an activated B cell phenotype and upregulation of costimulatory molecules. CD21^+^CD86^+^ in % of CD19^+^CD20^+^, representing recently activated B cells, occurred more frequently in patients with early relapse (7.9% ± 0.9) than in patients in long-term remission (5.8% ± 0.6, *p* < 0.05; Fig. 3b). Subpopulations with attributable immunostimulatory characteristics occurred in variable distribution between both groups: Disease-free patients had reduced CD40^+^ in % of CD19^+^ cells (85.1% ± 2.3 vs. 90.4% ± 1.2; *p* = 0.04) and lower proportions of BAFF-R^+^ in % of CD19^+^ (47.0% ± 7.9 vs. 67.8% ± 4.3; *p* = 0.03; Fig. 3b). Tim-3, which may mediate NK cell tolerance, was significantly upregulated on CD56^+^ and CD56^dim^ NK cells in patients with early relapse compared to patients with long-term remission (CD56^+^ NK cells: MFI 3.6 ± 0.3 vs. 2.7 ± 0.2, *p* = 0.01; CD56^dim^: MFI 3.7 ± 0.3 vs. 2.7 ± 0.2, *p* = 0.01; Fig. 3b). Proportions of activated NK-cells (CD25^+^%CD56^+^) did not differ between both groups (long-term remission 0.33% ± 0.12 vs. early relapse 0.20% ± 0.04; Supplementary Fig. 6h).

### Antigen-specific T cell responses after MWA are associated with longer disease-free survival

**Fig. 4:**
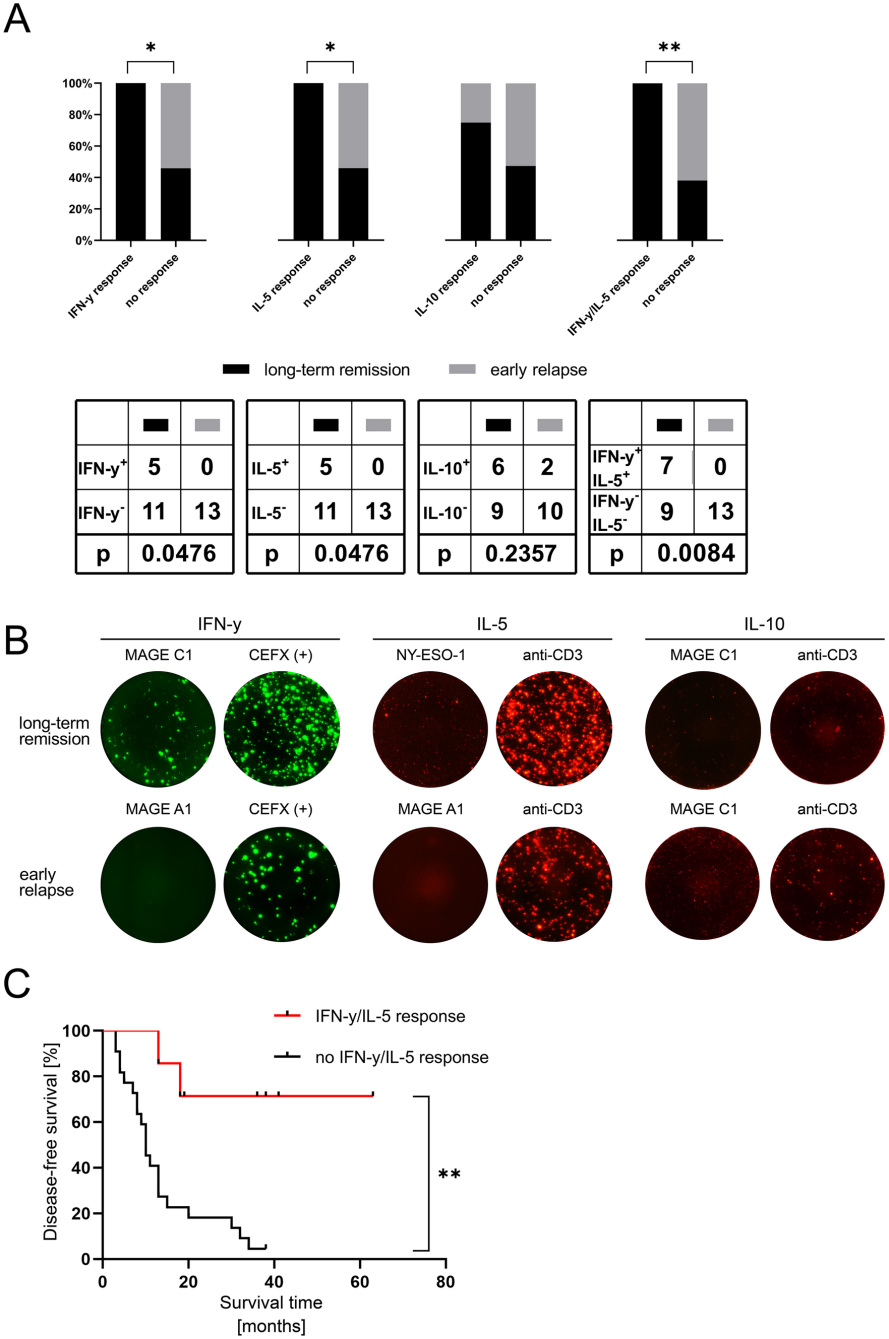
Analysis of the tumor antigen-specific T cell response by Fluorospot analysis: **a** comparison of the presence of tumor antigen-specific IFN-y, IL-5, IL-10 response after MWA in the two retrospective cohorts (*n* = 29). Panels show the percentage and absolute number of patients with TAA-specific cytokine response. **b** Exemplary Fluorospot analysis of IFN-y response against MAGE-A1 (long-term remission patient 2) and MAGE-C1 (relapsed patient 11), IL-5 response against NY-ESO-1 (long-term remission patient 2) and MAGE-A1 (relapsed patient 11) and IL-10 response against MAGE C1 (long-term remission patient 3) and MAGE C1 (relapsed patient 11). Positive controls (CEFX (+) for IFN-y or anti-CD3 for IL-5 and IL-10) for each patient are depicted on the right. **c** Kaplan Meyer analysis of disease-free survival of all retrospective patients (*n* = 29) depending on the presence of either IFN-y or IL-5 response against any CTA

We performed Fluorospot analyses of IFN-y, IL-5 and IL-10 responses induced by the seven above-mentioned HCC-associated antigens in patients with recurrence of disease within 1 year (*n* = 13) and patients experiencing long-term remission for more than 1 year (*n* = 16). 2 IL-10 samples were excluded due to failure of the positive control (Supplementary Fig. 4). We could not detect TAA-specific induction of IFN-y or IL-5 secretion in PBMC of any of the 13 relapsed patients. PBMC of two out of 12 patients in this group (16.7%) showed IL-10 secretion upon stimulation with TAAs, in particular for MAGE-C1 and PRAME. In contrast, HCC-associated antigens frequently elicited T cell-mediated immune responses in disease-free patients: 7/16 patients (43.75%) showed IFN-y or IL-5 secretion upon stimulation with TAAs (Fig. 4a and representative pictures 4b). Specific IFN-y or IL-5 responses to more than one TAA could be detected in 3 and 1 of these patients, respectively. We detected responses to all of the seven antigens: MAGE-A1, MAGE-C1 and MAGE-A4 were the antigens most frequently inducing IFN-y response (2/16 each) and NY-ESO-1 and MAGE-A1 were the predominant TAAs inducing IL-5 responses (2/16 each). Secretion of the immunosuppressive cytokine IL-10 was detected in 6/15 disease-free patients (40%). The frequencies of IFN-y and IL-5 responses were significantly increased in patients with long-term remission compared to patients with early relapse (Fig. 4a; *p* < 0.05 for IFN-y and IL-5, respectively). As previous treatments may represent a confounding variable, we compared TAA-specific immune responses in pretreated and non-pretreated patients. This comparison and also stratification of survival in the cohorts with long-term remission and early relapse did not show significant differences between pretreated and non-pretreated patients (Supplementary Fig. 5b and c). Stratification of patients with (*n* = 7) and without (*n* = 22) TAA-specific IL-5 or IFN-y response, revealed a correlation between HCC-associated antigen-specific T cell responses and disease-free survival (DFS; 27.5 vs. 10 months; *p* = 0.002; Fig. 4c).

### High T cell infiltration at the time of treatment is related to longer disease-free survival

**Fig. 5:**
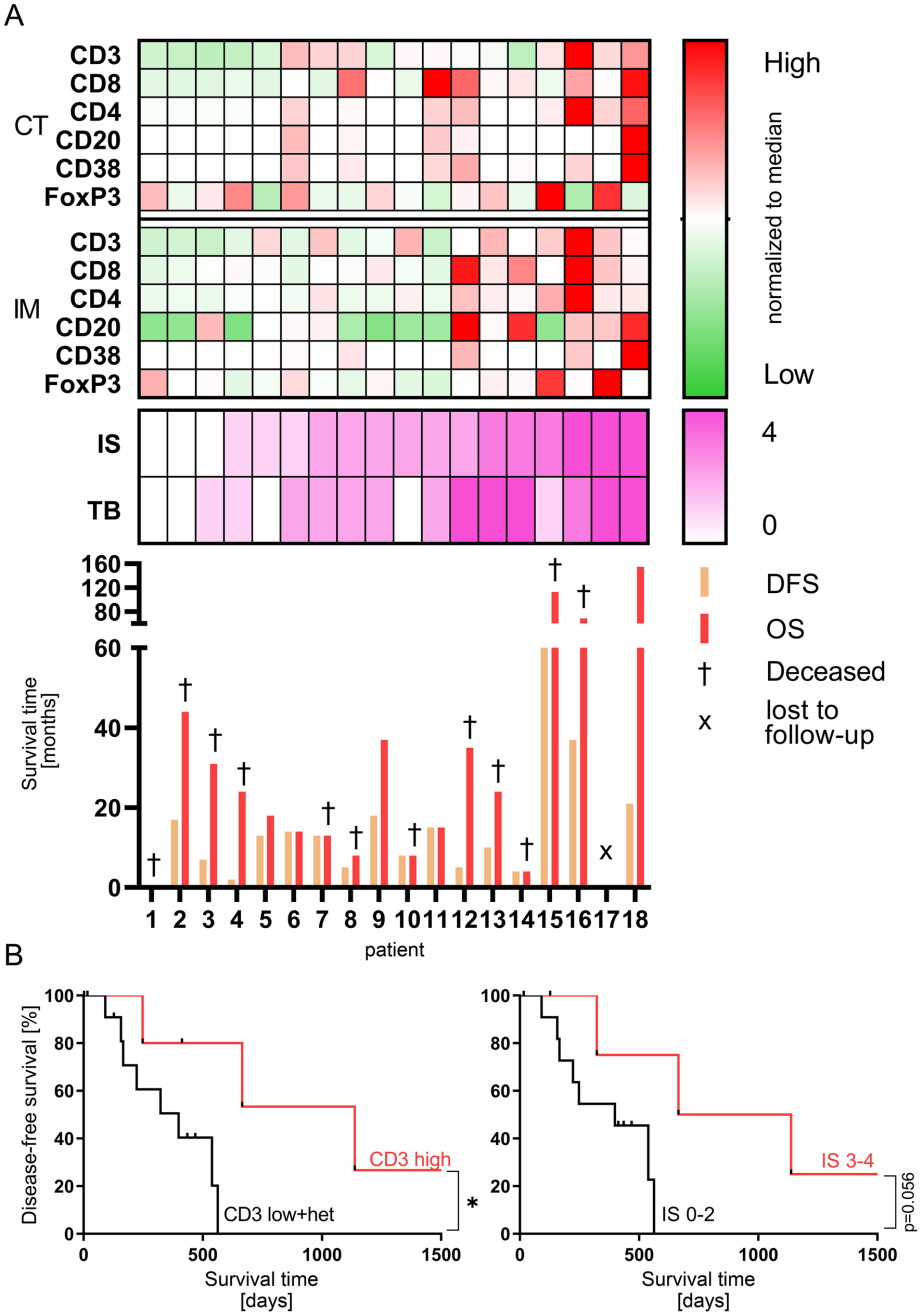
Immunohistochemical analysis of resected tumor tissues of patients with a combination therapy of thermoablation and surgical resection (*n* = 18). **a** Heatmap showing cell densities of CD3, CD8, CD4, CD20, CD38 and FoxP3 expressing cells in the center of the tumor (CT) and the invasive margin (IM) normalized to the median cell density of each marker for each region dividing them into either high (> median) or low (< median) infiltrated areas. Below, CD3/CD8 Immunoscore (IS), CD8/CD20 TB-Score (TB), overall (OS) and disease-free survival (DFS) are depicted for each patient. **b** Kaplan Meyer analysis of DFS depending on either high CD3 infiltrates (left) in CT and IM (CD3 high) or low CD3 infiltrates and heterogenous CD3 infiltrates in CT and IM (CD3 low + het) or rather depending on high IS (IS 3–4) or low IS (IS 0–2) (right)

To evaluate the impact of type and density of tumor-infiltrating lymphocytes on MWA in HCC patients, we analyzed resection specimens of patients receiving simultaneous MWA and surgical resection of different HCC lesions (*n* = 18). Tumor slides were immunohistochemically stained for CD3, CD8, CD4, CD20, CD38 and FoxP3. Based on the densities (cells/mm^2^) of CD3^+^ and CD8^+^ or CD3^+^ and CD20^+^ lymphocytes infiltrating the CT and IM, we determined the Immunoscore (IS) and the T and B cell score (TB Score) for each patient as an indirect parameter for the immunogenicity of individual tumors [21]. In our cohort of 18 patients, we observed a trend towards longer OS in patients with higher IS and TB Scores (Fig. 5a). We then classified patients into groups with high and low infiltration of CD3^+^, CD8^+^ and CD20^+^ lymphocytes, or with high (IS 3–4) and low IS (IS 0–2), respectively. Remarkably, we observed a significantly improved DFS in patients with high CD3^+^ T cell infiltration (13 vs. 37 months, *p* = 0.03), while values for DFS (*p* = 0.06) and OS (*p* = 0.24) did not show a difference between patients with high and low Immunoscores (Fig. 5b). Taken together, our data suggest that a high density of CD3^+^ T cells, but not CD8^+^ and CD20^+^ lymphocytes, in HCC lesions is related to superior outcome following MWA.

## Discussion

Immunogenic effects of local thermoablative therapies have been primarily described in the context of RFA. Only a few and mainly preclinical studies addressed the immune response following MWA in liver tissue [7, 8]. Detailed knowledge of immune-mediated effects of MWA is crucial to define the role of this treatment modality in the context of emerging immunotherapies [11, 25]. De novo and enhanced tumor-specific immune responses in our study demonstrate immunogenic anti-tumor effects of MWA in HCC. The effect of MWA on circulating immune cell subsets was moderate and mainly transiently detectable on day 7 after ablation. We identified higher proportions of CD3^+^ T cells and effector memory T cells as correlates of a remission lasting more than 1 year and an increase of Tim-3-expressing NK cells, which have been shown to inhibit anti-tumor immunity [26]. Two previous studies investigated changes in circulating immune cells and provided evidence for a weak but detectable immune response after MWA in HCC patients. In line with this data, circulating regulatory T cells as the major immunosuppressive T cell subset seem to be largely unchanged by MWA [19, 20]. B and NK cell subsets in PBMC of patients following MWA have not been investigated previously. Zhou et al. identified Th17 cells to be exclusively increased post treatment and determined a correlation of high baseline levels and the risk of tumor recurrence [19]. Zhang et al. found an increased proportion of T cells, particularly CD4^+^ T cells, and enhanced IL-12 secretion one month following MWA [20]. Taken together, we detected only moderate effects of MWA on circulating immune cell subsets and our results are in line with previous studies. An increase of circulating plasmablasts may indicate an impact of MWA on anti-tumor B cell response, which goes in line with recent data on the role of B cells in cancer immunotherapy [27]. Studies analyzing the impact of RFA on circulating immune cell subsets revealed comparably modest changes and the effects of MWA and RFA seem to be similar [15].

In addition to these phenotypic analyses, we investigated the tumor-specific T cell response against a set of TAAs. Tumor-specific endogenous CD8^+^ T cell responses have been described in HCC using IFN-y response upon stimulation with tumor cell lysates or by stimulation of PBMC with selected tumor-associated peptides or peptide pools in enzyme-linked immunosorbent (ELISPOT) assays [15, 17]. In our study, we used a 3-color Fluorospot assay allowing analyses of tumor-specific IFN-y, IL-5 and IL-10 producing lymphocytes. Compared to the reported rate of 15% of treatment-naïve patients showing tumor-specific T cell responses [15], endogenous IFN-y responses were 10% in our prospective cohort. To our knowledge there is no data describing tumor-specific IL-5 or IL-10 producing T cells in HCC as an indicator for an involvement of Th2 response in sufficient antitumor response as well as investigating possible immunosuppressive effects of MWA [28, 29]. Interestingly, we found 29.4% IL-10 but no IL-5 responses in the analyzed treatment-naïve patients. This is the first study describing tumor-specific immune responses following MWA treatment. Despite preclinical evidence of immune-mediated effects of thermal ablation, only very few studies investigated this aspect in samples from human cancer patients. Mizukoshi et al. described remarkable immune-related effects in a cohort of HCC patients. They used a set of 11 single peptides to detect immune responses against the five tumor-associated antigens SART2, SART3, MRP3, AFP and hTERT and described an RFA-induced increase of IFN-y producing tumor-specific T cells for all of these peptides. 62% of patients had increased IFN-y producing T cells following RFA. The percentages of patients with peptide-specific responses varied between 10 and 35%. We detected TAA-specific IFN-y or IL-5 secretion in 30% (6/20) of patients. Similar to the previously reported results, long-lasting T cell memory only emerges in a minority of patients [17]. We included a higher number of TAAs and used peptide pools (pools of peptides spanning the whole protein) instead of single peptides. Theoretically, the fraction of responding patients should be higher in our cohort. Lower immunogenicity of the cancer-testis antigens used in our protocol and different HCC etiology in Asian (Hepatitis) and Caucasian (mainly non-Hepatitis) patients are possible reasons for this observation [17]. Taken together, the frequency of anti-tumor immune responses induced by RFA and MWA seem to be comparable. The experimental strategy for monitoring tumor-specific immune response in HCC may be further improved by the inclusion of additional antigens. In addition to the described shared antigens, private neoantigens derived from somatic mutations could be of similar importance for immune-related effects of thermal ablation [30]. Given that these neoantigens, several additional cancer testis antigens and other TAAs were not investigated in both studies a higher frequency of endogenous tumor-specific immune responses can be expected in HCC. In another study, ELISPOT assays of T cells stimulated with autologous tumor cell lysates showed a response in 9/20 patients treated with RFA [15]. In summary, the diagnostic approaches applied in the two studies of RFA-treated and in our study with MWA-treated HCC patients showed an increase of tumor-specific immune response and highlight the immunogenic effect of thermoablation. This is of particular importance for monitoring of immune response in ongoing and future clinical trials of thermal ablation in combination with immunotherapy.

Whether enhanced tumor-specific T cell reactivity after RFA is correlated to longer progression-free survival (PFS) and OS is controversial. Zerbini et al. could not demonstrate longer PFS in patients with T cell responses to tumor cell lysates [15], while T cell response was associated to prolonged survival in the above-described study including 69 HCC patients [17]. We included an additional cohort comprising patients with early relapse (< 1 year) and long-term remission (> 1 year). Seven out of 16 (43.75%) patients with long-term remission had TAA-specific IFN-y or IL-5 responses, while none of the 13 patients with early relapse did. Moreover, DFS was longer for patients with TAA-specific IFN-y or IL-5 responses. Taken together, our data suggest an impact of tumor-specific immune response on disease control following MWA. This is the first study describing this in the context of MWA and our data is supported by the described findings in patients undergoing RFA. The relatively small number of included patients and their heterogeneity regarding clinical parameters have to be considered as major limitations of our study. Although we did not observe significant differences between the compared patient cohorts regarding tumor size, underlying disease or previous treatments (Table 1, Supplementary Fig. 5), these clinical parameters have to be considered as possible confounders. Future studies including a higher number of patients with strict inclusion criteria, probably in a multicenter setting, are needed to confirm our results.

A possible surrogate for immunogenicity of cancer are type, density and localization of tumor-infiltrating lymphocytes. Galon et al. used digital quantification of T cells in tissue microarrays of colorectal cancer patients and found a strong correlation of high T cell infiltration and superior prognosis [31]. They developed the “Immunoscore” as a standardized tool for digital quantification of T cell infiltrates [22]. Tumor-infiltrating cells reflect immunogenicity also in HCC, particularly high CD3^+^ and CD8^+^ cell infiltration was demonstrated to correlate with prolonged relapse-free survival [32]. We assumed that the immunogenicity of different HCC lesions in one individual is similar and identified a relation of CD3^+^, but not CD20^+^ abundance in resected tumor lesions to superior outcome following combined treatment with surgery and thermal ablation. Nevertheless, B cells could be of additional relevance considering recent studies on the role of B cells for response to checkpoint blockade [27]. Other aspects related to immunogenicity may be of similar importance and need further investigation [33]. Steel et al. recently demonstrated a worse prognosis for HCC patients with high mutational burden, which is in contrast to most other types of cancer [34–36]. The liver seems to be particularly efficient in induction of immunologic tolerance and alterations in MHC I expression. Altered processing or expression of (neo-)antigens and an immunosuppressive immune cell infiltrate have to be considered as potential obstacles for immunotherapy of HCC [25, 37, 38].

Taken together, the data presented in this study and the discussed previous results in the context of RFA demonstrate that MWA and RFA induce remarkable local and systemic immune-stimulatory effects [4, 11, 18]. Combination of MWA and immune checkpoint inhibition might augment the preliminary success of checkpoint inhibition in this disease [39–41].

## Electronic supplementary material

Below is the link to the electronic supplementary material.

Supplementary file1 Supplementary Table 1. Clones and dilutions of antibodies for immunohistochemistry and 10-color flow cytometry. (PDF 141 kb)

Supplementary file2 Supplementary Figure 1. Exemplary images showing placement of MWA needle, measurement of tumor and ablation diameters and calculation of ablation:tumor ratio. (PDF 779 kb)

Supplementary file3 Supplementary Figure 2. Gating strategy for flow cytometric analyses. (PDF 1502 kb)

Supplementary file4 Supplementary Figure 3. Digital image analysis of immunohistochemical stained resected HCC samples. (A) cross sections of HCC stained for CD3, CD8, CD20, CD38, CD4 and FoxP3 are subjected to automated analysis of IM and CT. IM (area ranging from 50μm within the tumor to 300μm outside the tumor border) and CT (whole tumor section excluding first 50 μm adjacent to the tumor border) are schematically shown on the left side. Both areas are subsequently separated and cropped into tiles suitable for automated analysis of AEC-stained cells. (B) Exemplary tiles derived from IM and CT stained for CD3 before and after analysis (positive cells are displayed in green, nuclei in yellow). As a result, cell count per mm^2^ is calculated for the whole area of IM and CT, respectively. (PDF 2694 kb)

Supplementary file5 Supplementary Figure 4. Presentation of positive controls of 3-Colour-Fluorospots. (A) Heatmap showing an overview of IFN-y responses against CEFX peptide pool (biological positive control), and IFN-y, IL-5 and IL-10 secretion after stimulation with an anti-CD3 antibody as technical positive control for prospective patients and (B) for retrospective cohorts. Samples with failure of positive control were excluded from results (prospective: 9 IL-10 samples, 2 IL-5 samples; retrospective: 2 IL-10 samples). (PDF 719 kb)

Supplementary file6 Supplementary Figure 5. (A) Characteristics of previous treatments. (B) Patients of the retrospective cohort were stratified into pretreated and non-pretreated patients and examined for different diseasefree survival after MWA using Kaplan Meier analysis. Left: long-term remission (left, *p*=0.78). Right: early relapse (right, *p*=0.27). (C) Comparison of tumor-specific immune response in pretreated (*n*=20) and non-pretreated (*n*=29) patients included in our study. (PDF 1183 kb)

Supplementary file7 Supplementary Figure 6. NK cells in PBMCs of MWA patients (n = 20) were examined by flow cytometry for alterations of the NK cell subpopulations of CD56dim and CD56bright NK cells (A), of activated NK cells (B) and for the expression of the immunoinhibitory molecules NKG2A (C), CD158b (D), CD158k Microwave ablation enhances tumor-specific immune response in patients with hepatocellular carcinoma (Leuchte K, Staib E, Thelen M, et al.). (E), CD158a (F) on day d0, d7, d90. NK cells in PBMCs of MWA patients with early relapse and long-term remission were examined by flow cytometry for the distribution of CD56dim and CD56bright NK cells (G), for activation (CD25+%CD56+) and expression of immunoinhibitory NKG2A, CD158b, CD158k, CD158a (H) and the expression of Tim-3 (I). (PDF 464 kb)

## Data Availability

The datasets generated and analyzed during the current study are available from the corresponding author, KL, upon reasonable request.

## Abbreviations

CT: Center of the tumor
DFS: Disease-free survival
DNAM: DNAX Accessory Molecule-1
IM: Invasive margin
MWA: Microwave ablation
NK cells: Natural Killer cells
OS: Overall survival
PBMC: Peripheral blood mononuclear cells
RFA: Radiofrequency ablation
TAA: Tumor-associated antigen
