# Supplementary material for: Microwave ablation enhances tumor-specific immune response in patients with hepatocellular carcinoma
Source: Cancer Immunol Immunother. 2020 Oct 2;70(4):893–907. doi: 10.1007/s00262-020-02734-1 (PMC7979675; doi:10.1007/s00262-020-02734-1)
Supplement: Supplementary file 1 — Supplementary file1 Supplementary Table 1. Clones and dilutions of antibodies for immunohistochemistry and 10-color flow cytometry. (PDF 141 kb) [file 262_2020_2734_MOESM1_ESM.pdf]

Epitop	Host	Company, Catalog Number	Isotype	Dilution
CD3	rabbit	LS Bio LS-B8669	IgG	1:100
CD8	mouse	Dianova DLN-009534	IgG1	1:50
FoxP3	mouse	Abcam ab20034	IgG1	1:50
HLA-ABC	mouse	Abcam ab70328	IgG1	1:100
CD20	mouse	Zytomed MSK008-05	IgG2a	1:100
CD4	rabbit	Abcam ab133616	IgG	1:100
CD38	mouse	Novocastra NCL-L-CD38-290	IgG1	1:100

Fluorochrome	Target	Clone	Company	Catalogue No.
FITC	CD45	HI30	Biolegend	304038
PE	CD273 (PD-L2)	MIH18	Biolegend	345506
PE	CD226 (DNAM-1)	11A8	Biolegend	338306
PE	CD270 (HVEM)	122	Biolegend	318806
PE	CD258 (LIGHT)	T5-39	Biolegend	318706
PE	CD48	BJ40	Biolegend	336708
PE	CD197 (CCR7)	G043H7	Biolegend	353204
PE	CD152 (CTLA-4)	BNI3	BD Bioscience	555853
PE	CD69	FN50	Biolegend	310906
PE	CD86	IT2.2	Biolegend	305406
PE	CD158k	539304	R&D Systems	FAB2878P
PE-Dazzle	CD134 (OX40)	Ber-ACT35	Biolegend	350020
PE-Dazzle	CD155 (PVR)	SKII.4	Biolegend	337616
PE-Dazzle	CD154 (CD40L)	24-31	Biolegend	310840
PE-Dazzle	CD366 (Tim-3)	F38-2E2	Biolegend	345034
PE-Dazzle	CD244 (2B4)	C1.7	Biolegend	329522
PE-Dazzle	CD185 (CXCR5)	J252D4	Biolegend	356928
PE-Dazzle	CD25	M-A251	Biolegend	356126
PE-Dazzle	CD28	CD28.2	Biolegend	302942
PE-Dazzle	IgD	IA6-2	Biolegend	348240
PerCP-Cy5.5	CD40	5C3	Biolegend	334316
PerCP-Cy5.5	CD268 (BAFF-R)	11C1	Biolegend	316918
PerCP-Cy5.5	CD160	BY55	Biolegend	341210
PerCP-Cy5.5	Galectin-9	9M1-3	Biolegend	348910
PerCP-Cy5.5	CD305 (LAIR1)	NKTA255	Biolegend	342804
PerCP-Cy5.5	CD8	SK1	Biolegend	344710
PerCP-Cy5.5	CD127	A019D5	Biolegend	351322
PerCP-Cy5.5	CD24	ML5	Biolegend	311116
PerCP-Cy5.5	CD158b	DX27	Biolegend	312614
PE-Cy7	CD56	5.1H11	Biolegend	362510
PE-Cy7	CD45RA	HI100	Biolegend	304126
PE-Cy7	CD73	A2D	Biolegend	344010
PE-Cy7	CD27	O323	Biolegend	302838
Alexa Fluor 647	CD279 (PD1)	EH12.1	BD Bioscience	560838
Alexa Fluor 647	FoxP3	259D	Biolegend	320214

APC	CD96 (TACTILE)	REA195	Miltenyi	130-101-028
APC	CD159a (NKG2A)	REA110	Miltenyi	130-113-563
APC	CD66 ace	ASL-32	Biolegend	342308
APC	VISTA	730804	R&D Systems	FAB71261A
APC	CD21	Bu32	Biolegend	354906
APC	CD178	NOK-1	Miltenyi	130-096-458
Alexa Fluor 700	CD3	SK7	Biolegend	344822
Alexa Fluor 700	CD20	2H7	Biolegend	302322
APC-Fire 750	CD19	SJ25C1	Biolegend	363030
APC-Fire 750	CD4	SK3	Biolegend	344638
APC-Fire 750	CD8	SK1	Biolegend	344746
BV421	CD274 (PD-L1)	29E.2A3	Biolegend	329714
BV421	TIGIT	A15153G	Biolegend	372710
BV421	CD272 (BTLA)	MIH26	Biolegend	344512
BV421	CD223 (LAG3)	11C3C65	Biolegend	369314
BV421	CD357 (GITR)	108-17	Biolegend	371208
BV421	CD278 (ICOS)	C398.4A	Biolegend	313524
BV421	CD39	A1	Biolegend	328214
BV421	CD137 (4-1BB)	4B4-1	Biolegend	309820
BV421	CD38	HIT2	Biolegend	303526
BV421	CD158a	HP-3E4	BD Bioscience	564318
Zombie Aqua	fixable viability dye		Biolegend	423102
