# Supplementary figures and images for: Microwave ablation enhances tumor-specific immune response in patients with hepatocellular carcinoma
Source: Cancer Immunol Immunother. 2020 Oct 2;70(4):893–907. doi: 10.1007/s00262-020-02734-1 (PMC7979675; doi:10.1007/s00262-020-02734-1)

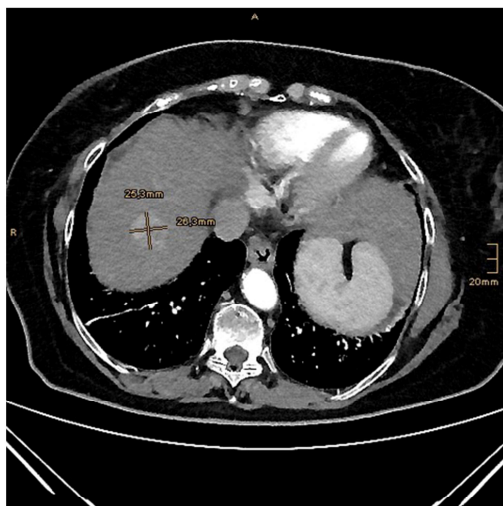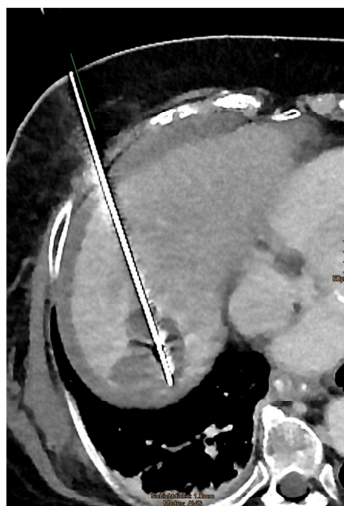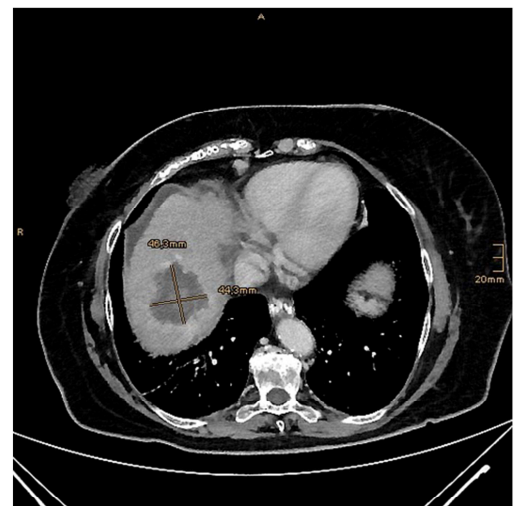

Calculation of ablation:tumor ratio:

$$d_{\text{max Ablation}} / d_{\text{max Tumor}}$$

Supplement: Supplementary file 2 — Supplementary file2 Supplementary Figure 1. Exemplary images showing placement of MWA needle, measurement of tumor and ablation diameters and calculation of ablation:tumor ratio. (PDF 779 kb) [file 262_2020_2734_MOESM2_ESM.pdf]

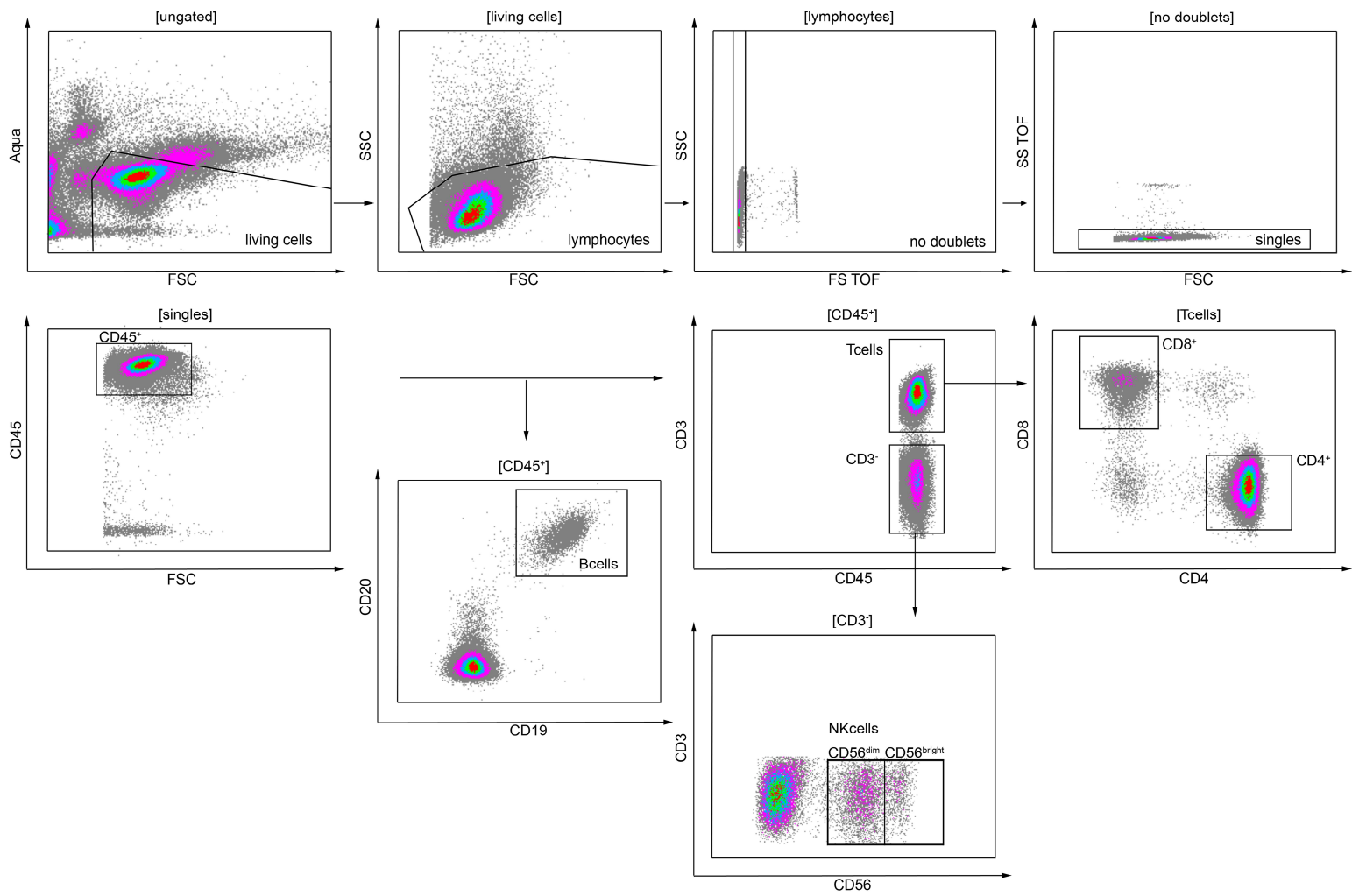

Supplement: Supplementary file 3 — Supplementary file3 Supplementary Figure 2. Gating strategy for flow cytometric analyses. (PDF 1502 kb) [file 262_2020_2734_MOESM3_ESM.pdf]

A

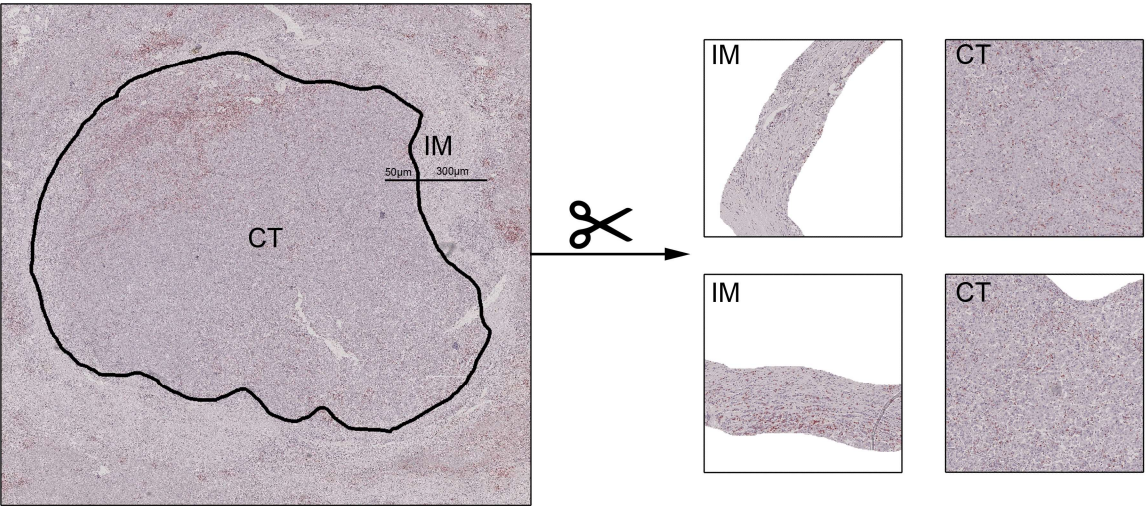

B

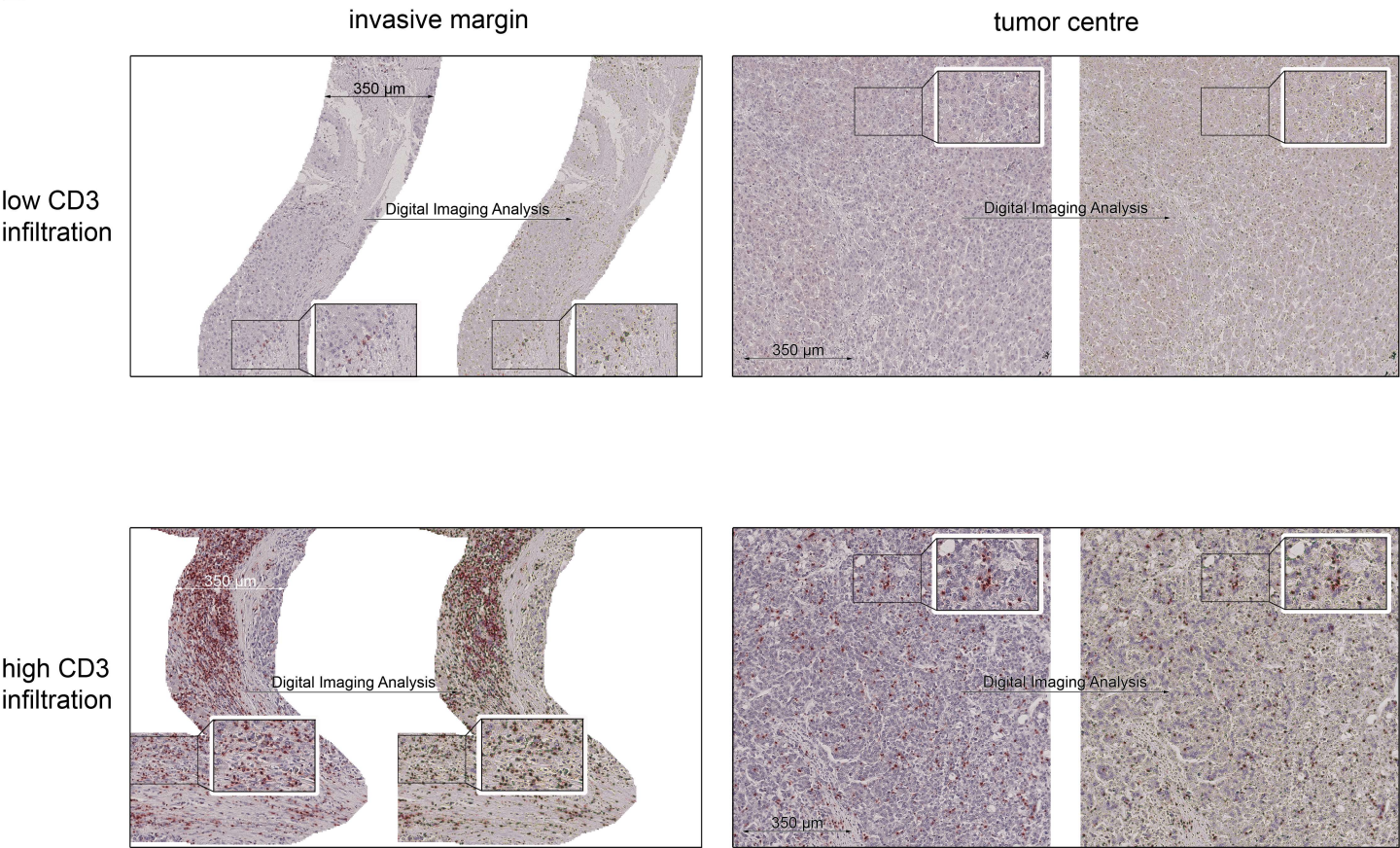

Supplement: Supplementary file 4 — Supplementary file4 Supplementary Figure 3. Digital image analysis of immunohistochemical stained resected HCC samples. (A) cross sections of HCC stained for CD3, CD8, CD20, CD38, CD4 and FoxP3 are subjected to automated analysis of IM and CT. IM (area ranging from 50μm within the tumor to 300μm outside the tumor border) and CT (whole tumor section excluding first 50 μm adjacent to the tumor border) are schematically shown on the left side. Both areas are subsequently separated and cropped into tiles suitable for automated analysis of AEC-stained cells. (B) Exemplary tiles derived from IM and CT stained for CD3 before and after analysis (positive cells are displayed in green, nuclei in yellow). As a result, cell count per mm2 is calculated for the whole area of IM and CT, respectively. (PDF 2694 kb) [file 262_2020_2734_MOESM4_ESM.pdf]

A

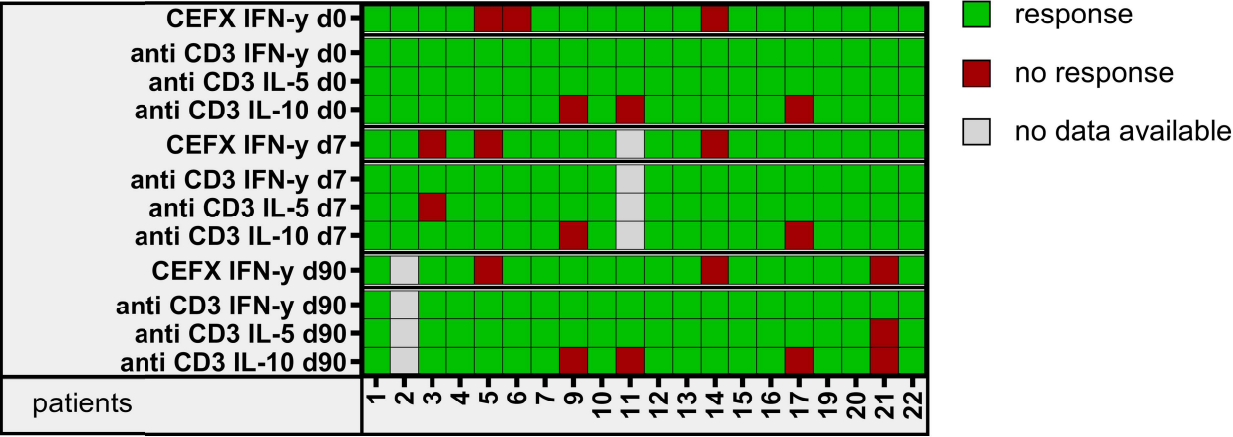

B

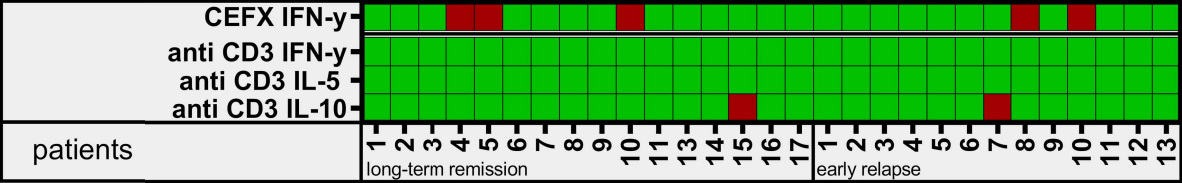

Supplement: Supplementary file 5 — Supplementary file5 Supplementary Figure 4. Presentation of positive controls of 3-Colour-Fluorospots. (A) Heatmap showing an overview of IFN-y responses against CEFX peptide pool (biological positive control), and IFN-y, IL-5 and IL-10 secretion after stimulation with an anti-CD3 antibody as technical positive control for prospective patients and (B) for retrospective cohorts. Samples with failure of positive control were excluded from results (prospective: 9 IL-10 samples, 2 IL-5 samples; retrospective: 2 IL-10 samples). (PDF 719 kb) [file 262_2020_2734_MOESM5_ESM.pdf]

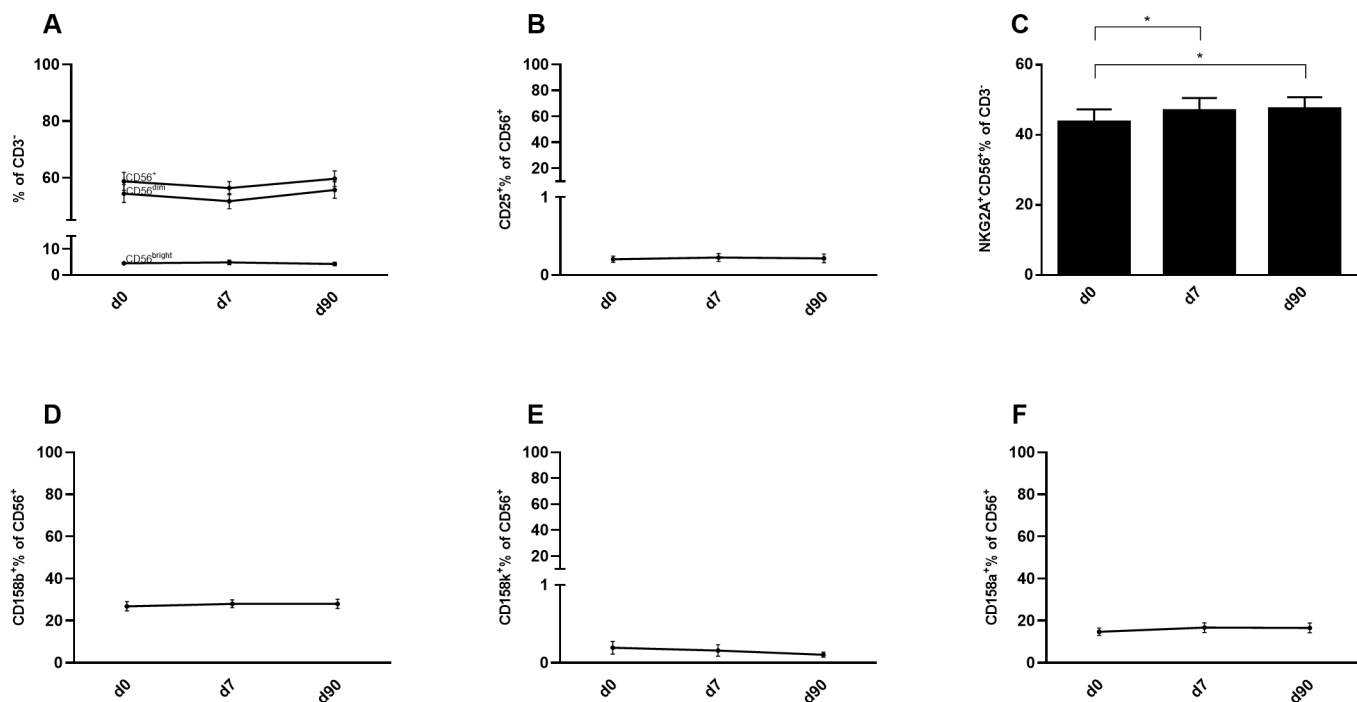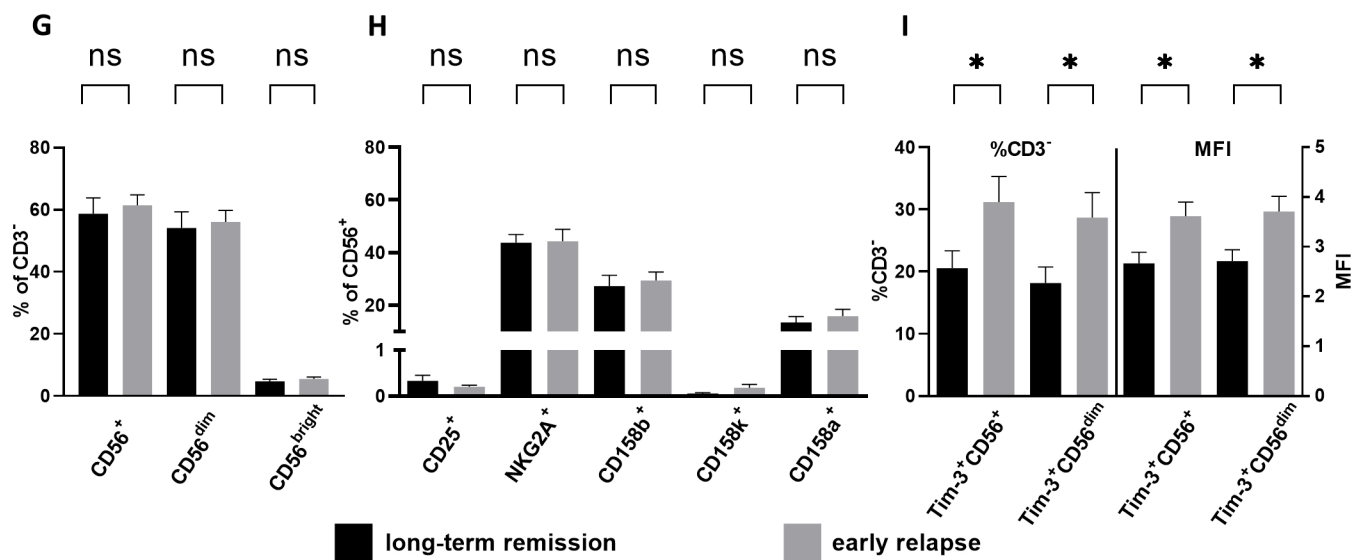

Supplement: Supplementary file 7 — Supplementary file7 Supplementary Figure 6. NK cells in PBMCs of MWA patients (n = 20) were examined by flow cytometry for alterations of the NK cell subpopulations of CD56dim and CD56bright NK cells (A), of activated NK cells (B) and for the expression of the immunoinhibitory molecules NKG2A (C), CD158b (D), CD158k Microwave ablation enhances tumor-specific immune response in patients with hepatocellular carcinoma (Leuchte K, Staib E, Thelen M, et al.). (E), CD158a (F) on day d0, d7, d90. NK cells in PBMCs of MWA patients with early relapse and long-term remission were examined by flow cytometry for the distribution of CD56dim and CD56bright NK cells (G), for activation (CD25+%CD56+) and expression of immunoinhibitory NKG2A, CD158b, CD158k, CD158a (H) and the expression of Tim-3 (I). (PDF 464 kb) [file 262_2020_2734_MOESM7_ESM.pdf]
