# Supplementary material for: Microwave ablation enhances tumor-specific immune response in patients with hepatocellular carcinoma
Source: Cancer Immunol Immunother. 2020 Oct 2;70(4):893–907. doi: 10.1007/s00262-020-02734-1 (PMC7979675; doi:10.1007/s00262-020-02734-1)

A

	Patient number	Number of pretreatments	Type of pretreatment	date of pretreatment	time interval to investigated MWA (days)
Prospective Patients	5	1	RFA	12/2015	580
	6	1	MWA	04/2016	472
	7	1	MWA	06/2016	310
	10	1	surgical resection	01/2017	226
	14	1	surgical resection	04/2017	215
	16	1	TAE	12/2016	119
	17	3	3 x TACE	12/2014	687
				02/2015	
				03/2015	
	20	1	RFA	06/2016	588
	23	1	surgical resection	06/2017	289
Relapsed Patients	5	1	RFA	12/2015	580
	3	3	surgical resection	07/2013	200
			TACE	07/2013	
			surgical resection	11/2013	
	8	1	stereotactic radiotherapy	07/2015	406
	9	1	surgical resection	05/2015	389
	11	4	RFA	11/2011	441
			RFA	2013	
			Gammaknife	07/2015	
			MWA	03/2016	
	12	1	TAE	12/2016	119
	13	3	TACE	12/2014	687
			TACE	02/2015	
			TACE	03/2015	
Patients in Long-term Remission	17	1	MWA	03/2014	907
	4	1	RFA	02/2013	753
	9	2	surgical resection	2013	
			RFA	02/2013	527
	11	3	RFA	11/2011	252
			RFA	03/2013	
			Cyberknife	02/2015	
	14	1	MWA	03/2015	77
Mean					420

B

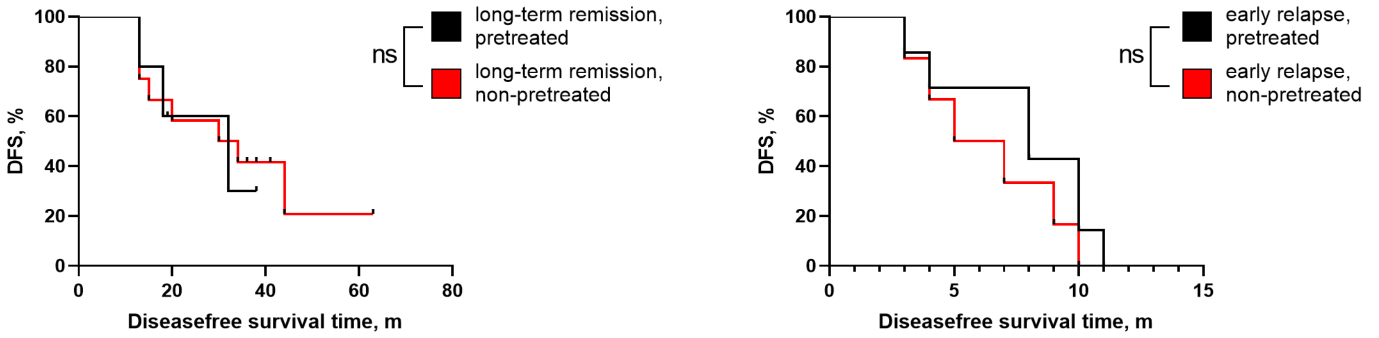

C

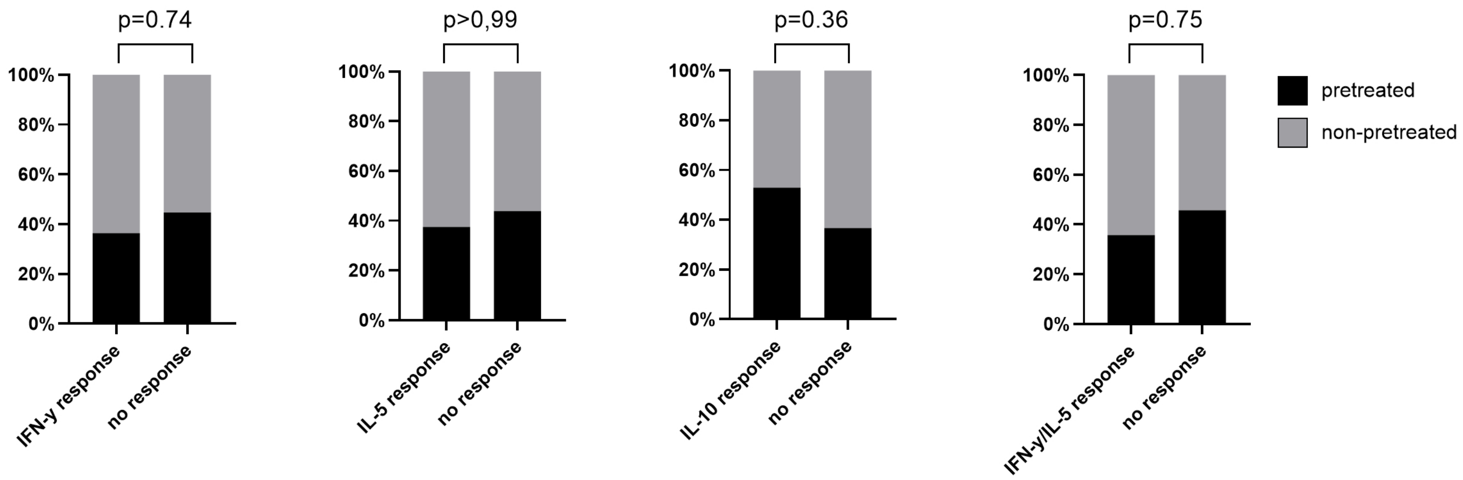

Supplement: Supplementary file 6 — Supplementary file6 Supplementary Figure 5. (A) Characteristics of previous treatments. (B) Patients of the retrospective cohort were stratified into pretreated and non-pretreated patients and examined for different diseasefree survival after MWA using Kaplan Meier analysis. Left: long-term remission (left, p=0.78). Right: early relapse (right, p=0.27). (C) Comparison of tumor-specific immune response in pretreated (n=20) and non-pretreated (n=29) patients included in our study. (PDF 1183 kb) [file 262_2020_2734_MOESM6_ESM.pdf]
